# Imaging of Electrode Position after Cochlear Implantation with Flat Panel CT

**DOI:** 10.5402/2012/728205

**Published:** 2012-12-04

**Authors:** Diana Arweiler-Harbeck, Christoph Mönninghoff, Jens Greve, Thomas Hoffmann, Sophia Göricke, Judith Arnolds, Nina Theysohn, Ulrich Gollner, Stephan Lang, Michael Forsting, Marc Schlamann

**Affiliations:** ^1^Department of Otorhinolaryngology, University Hospital Essen, University of Duisburg-Essen, 45122 Essen, Germany; ^2^Department of Radiology and Neuroradiology, University Hospital Essen, University of Duisburg-Essen, 45122 Essen, Germany

## Abstract

*Background*. Postoperative imaging after cochlear implantation is usually performed by conventional cochlear view (X-ray) or by multislice computed tomography (MSCT). MSCT after cochlear implantation often provides multiple metal artefacts; thus, a more detailed view of the implant considering the given anatomy is desirable. A quite new method is flat panel volume computed tomography. The aim of the study was to evaluate the method's clinical use. *Material and Methods*. After cochlear implantation with different implant types, flat panel CT scan (Philips Allura) was performed in 31 adult patients. Anatomical details, positioning, and resolution of the different electrode types (MedEL, Advanced Bionics, and Cochlear) were evaluated interdisciplinary (ENT/Neuroradiology). *Results*. In all 31 patients cochlear implant electrode array and topographical position could be distinguished exactly. Spatial resolution and the high degree of accuracy were superior to reported results of MSCT. Differentiation of cochlear scalae by identification of the osseous spiral lamina was possible in some cases. Scanning artefacts were low. *Conclusion*. Flat panel CT scan allows exact imaging independent of implant type. This is mandatory for detailed information on cochlear electrode position. It enables us to perform optimal auditory nerve stimulation and allows feed back on surgical quality concerning the method of electrode insertion.

## 1. Introduction

Postoperative imaging after cochlear implantation usually is performed by conventional cochlear view (X-ray) or by multislice computed tomography (MSCT). Conventional cochlear view is routinely used mainly in children due to short investigation time and low radiation dose. This technique only gives projective information on the fact that insertion into the cochlea has been successful, but analysis of exact electrode position with regard to the topography of the cochlea is impossible [1, 2]. MSCT after cochlear implantation allows for three-dimensional imaging; however, it unfortunately provides metal artefacts; thus, a more detailed view of the electrodes with regard to the given anatomical structures is desirable [3–5]. This is not only of major importance for quality control as far as surgical insertion methods are concerned but also with regard to special anatomic situations (e.g., mondini dysplasia, ossification of cochlea due to meningitis or otosclerosis) and fitting conditions dependent on electrode array position and results of neural response telemetry in rehabilitation. In order to gain more information on perfect design and function of electrodes, an exact way of imaging also is mandatory from researchers' and developers' point of view.

An additional imaging method is flat-panel computed tomography. The experimental use of flat-panel high-resolution CT in temporal bone specimens was evaluated with regard to position of electrode array and with special concern on documentation of the highest possible accuracy in cranial base navigation [3, 6, 7].

The aim of our study was to evaluate the clinical use of flat-panel CT as far as feasibility, artefacts, position within the cochlea and the temporal bone, and visualization of electrodes of different implant types are concerned.

## 2. Materials and Methods

### 2.1. Subjects

Between December 2009 and September 2010 31 subsequently implanted adult patients (female = 11, male = 20; mean age 52 years) without inner ear malformations were included in this study one day after surgery. All received preoperative diagnostic imaging by CT scan and/or magnetic resonance imaging. 3 different cochlear implant devices were applied: Cochlear Nucleus CI 512 (*n* = 24) with 22 electrodes distributed over 15 mm, Advanced Bionics Implant Hi Res 90 (*n* = 4) with 16 electrodes distributed over 16.5 mm, and MedEl Sonata Ti (*n* = 3) with 12 pairs of electrodes with a distance of 2.4 mm each. Cochlear implantation was performed by the same surgeon; full insertion according to provided insertion depth was possible without complications in all cases.

### 2.2. Imaging

Flat-panel CT examinations were performed on a Philips Allura C-arc angiographic unit (Philips Medical Systems, Best, The Netherlands) connected to a 3DRA workstation (Philips Medical Systems).

With patients temporal bone in system isocenter, the scan was performed with a propeller movement covering 207° of the circular trajectory. 622 frames were exposed during the 20.7 s scan (30 frames/s), utilizing a detector format of 33 × 40 cm. Total examination time including bedding of the patient on the examination table demanded less than 2 minutes. 

Source images were transferred to the workstation during and after the rotational acquisition, and a volume data set was created. The reconstruction appeared on the workstation monitor. 

Multiplanar MIP reconstructions parallel to the cochlea were performed with a slice thickness of 1.5 mm and orthogonal to the cochlea with a slice thickness of 0.41 mm.

### 2.3. Evaluation/Analysis

Images were analysed by two independent investigators (otorhinolaryngology 1, neuroradiology 1). Artefacts were characterised between very low (= 1) and very high (= 6). Identification of scala tympani, scala vestibule, and osseous spiral lamina as well as tip fold over, full insertion, identification of distance between electrode array and modiolus (next to (n) and far from (f)), and possibility of identification of single electrodes within the electrode array was of further interest. 

## 3. Results

An overview on all subjects is illustrated in Table 1. In all cases, full insertion of all electrodes could be seen, which was in line with the surgeon's information. There was no tip fold over in any subject. The level of artefacts was calculated as 2.41 (1–6) in the mean for investigator I and 2.64 (1–6) for investigator II. Osseous spiral lamina could be identified in all subjects; scalae tympani and vestibuli were obviously visible in 6 of 31 cases (Figure 4). Differentiation of different types of implants was clearly feasible (Figures 2(a)–2(c)). Additional identification of single electrodes was possible in all implants of Medel and Advanced Bionics Companies. Identification was not always possible in Cochlear Nucleus implants due to artefacts produced by narrower distances between electrodes. Dependening on implant type, electrode array insertion could apparently be identified as next to modiolus (Figures 3(a) and 3(b)) in 25 cases or far from modiolus (Figures 3(c) and 3(d)) in 6 cases.

## 4. Discussion

**Figure 1:**
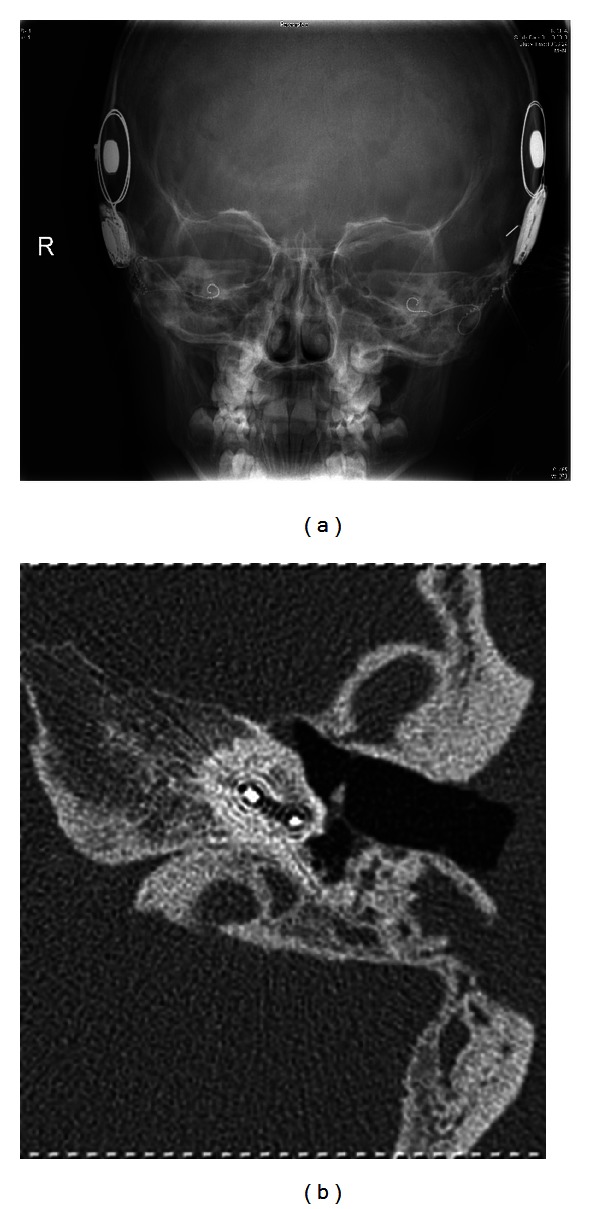
(a) Conventional X-ray cochlear view, (b) conventional multislice computed tomography.

**Figure 2:**
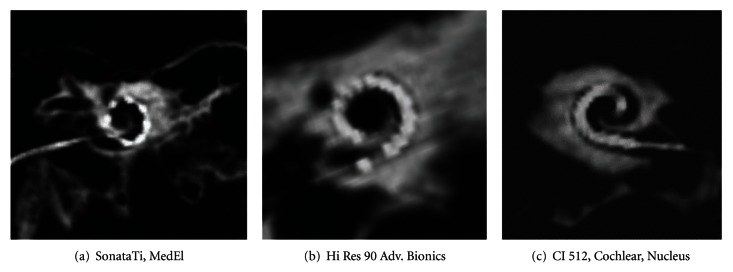
(a)–(c) Differentiation of single electrodes in diverse implant types.

**Figure 3:**
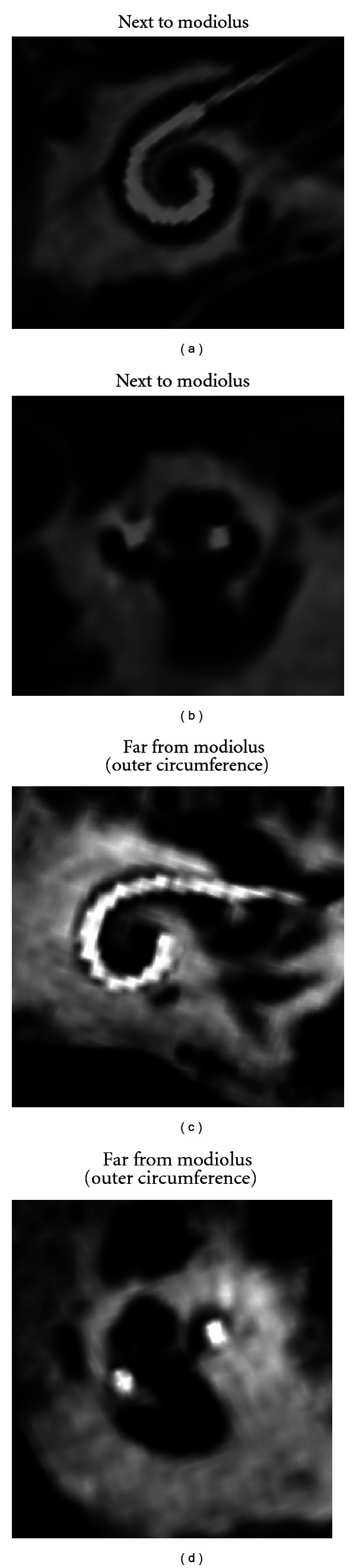
(a)–(d) Identification of electrode array position with regard to modiolus and cochlear nerve fibers.

**Figure 4:**
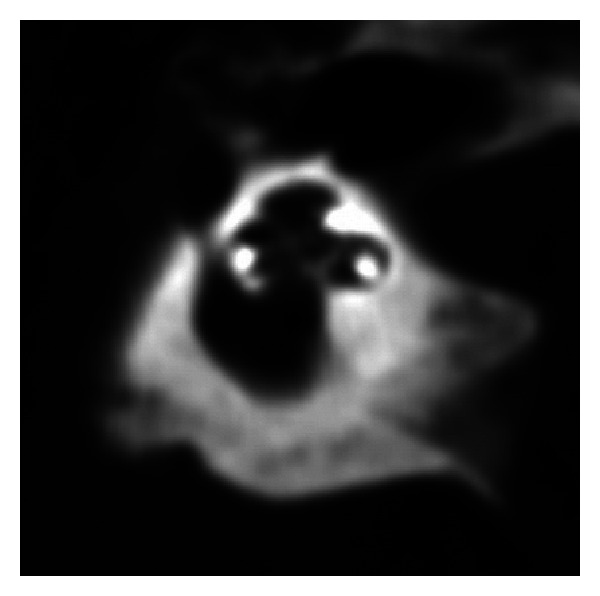
Visualization of scala tympani, scala vestibule, and osseous lamina spiralis.

Radiological examinations after cochlea implantation are mostly performed by conventional X-ray (Figure 1(a)). This method offers information about the position of the device within the petrous bone. Positioning within the cochlea is approximately indirectly educible from the configuration of the electrode. Additionally, buckling of the electrode may be visible. In more complex cases, a more detailed view of the electrode is eligible. In these cases, conventional MSCT scans offer additional information of the electrode positioning within the cochlea (Figure 1(b)). Nevertheless, conventional CT scans are often affected by artefacts due to electrode and generator [3–5]. 

Several attempts have been made to improve imaging. The technique of cone beam computed tomography as a low-dose imaging technique for postoperative assessment of cochlear implantation, which was tested in postoperative patients, seems to be promising with fewer artefacts and higher resolution than multislice CT [8]. But cone beam CT devices are still rare. Therefore, we evaluated the clinical impact of flat-panel CT in a C-arm angiography unit. 

Several publications deal with image quality of isolated temporal bone specimens [3] in flat-panel CT. They all revealed superior quality compared to multislice computed tomography. 

Rotational computed tomography (RT) is based on three-dimensional digital subtraction angiography. Images are taken with a rotating C-arm in a single rotation [9]. After digital reconstruction, the intracochlear position of an electrode array can be identified [10]. The method offers new possibilities in postsurgical imaging of cochlea implantation. Blooming artefacts are also less comprehensive, and spatial resolution is superior in comparison to MSCT [11, 12]. Due to higher spatial resolution, additional information of the position of the electrode in relation to the modiolus is offered. This has impact on the adjustment of the generator and the estimated life time of the battery. 

Of course, tip fold over should be clearly visible. In our collective, no patient had one. 

Electrode positioning within the scala tympani is clinically eligible. Otherwise, in case of positioning within the scala vestibuli, avoidable side effects like high impedances, reduced speech reception, and vertigo might occur due to damage of the intracochlear structures [13]. 

Exact positioning within cochlea was not assessable in our collective. As far as the literature is concerned, the assessment of positioning within the scala tympani most often could only be demonstrated in *ex vivo* specimens. 

Aschendorff et al. [9, 10] reported feasibility of assessing exact electrode position within the scala tympani or vestibuli *in vivo* by means of flat-panel CT. Scala media could not be visualized directly, and the position was concluded indirectly from curved reformatted images of the cochlea. In spite of similar examination techniques, the difference in the results might be based on different postprocessing. There is no possibility to perform curved reformations techniques which is impossible on our system. 

Some examinations have been performed to adjust radiation dose of FD CT in comparison to multislice CT. Radiation dose of FD CT is described to be lower than in MSCT. 

The main weak point of our study is the lack of direct comparison of MSCT and flat-panel CT. Due to the high level of artefacts known from own experience, MSCT is not common in our house under this indication. 

Our results indicate that flat-panel CT is a fast and accurate examination in the postoperative imaging of cochlear implants. It is of course superior to conventional X-ray, but it is also superior to MSCT mainly due to fewer artefacts. Additionally, radiation dose is lower than in MSCT. 

## Figures and Tables

**Table 1:** Patients and evaluation parameters of imaging.

Code	Age	Implant type	Artefact (1–6)		Full insertion	Tip fold over	Separation of electr.	Facial nerve	Differentiation of scalae
			Inv I/Inv II						
PW49	60	CN	4	3	+	−	−	?	−
KE36	73	CN	2	2	+	−	−	+	−
MG57	52	CN	2	3	+	−	−	?	−
LE46	63	CN	3	3	+	−	−	?	−
PB42	67	CN	2	2	+	−	−	+	−
WF52	57	ME	3	2	+	−	+	+	−
DM77	32	ME	4	3	+	−	+	?	−
UA74	35	CN	2	3	+	−	−	?	−
PU47	62	CN	3	3	+	−	−	?	−
OG57	52	AB	3	3	+	−	+	+	+
DU62	47	ME	2	2	+	−	+	−	−
BN77	33	CN	2	2	+	−	−	+	−
OK53	57	CN	3	4	+	−	−	+	−
KD67	43	CN	2	2	+	−	−	?	−
FM50	60	AB	3	3	+	−	+	−	−
WM65	45	CN	2	2	+	−	−	+	−
DR62	48	CN	2	2	+	−	−	+	−
LS71	39	CN	3	4	+	−	−	+	−
SP67	43	CN	2	3	+	−	−	+	−
BR63	47	AB	2	3	+	−	−	+	−
GH50	60	CN	3	3	+	−	+	−	−
ÖT67	43	CN	1	2	+	−	−	+	+
SM39	71	CN	2	3	+	−	−	?	−
HA51	59	CN	3	4	+	−	−	+	−
CE39	71	CN	2	3	+	−	−	+	−
WJ76	34	CN	3	3	+	−	−	?	−
GJ62	48	CN	4	4	+	−	−	+	−
ZT56	54	CN	1	2	+	−	−	−	+
TK75	35	CN	2	2	+	−	+	+	−
WJ40	70	AB	2	2	+	−	+	?	−
PD88	22	CN	1	2	+	−	−	?	+

Implant type: CN: Cochlear Nucleus CI512; ME: Medel Sonata Ti; AB: Advanced Bionics Hi Res 90.

Artefacts: 1: no artefacts and 6: evaluation impossible due to multiple artefacts.

Inv. I, II: investigator 1, investigator 2.

Full insertion +: all electrodes within cochlea; −: one or more electrodes outside cochlea.

Tip fold over +: tip of electrode is folded in the apical part; −: tip of electrode array is straight.

Separation of electrodes: +: single electrodes are clearly visible; −: no differentiation of single electrodes due to artefacts.

Position of facial nerve in projection to cochlea: +: clearly visible; −: not possible.

Differentiation of scalae tympani and vestibuli: +: clearly visible; /not possible.

Visualization of osseous lamina spiralis: +: clearly visible; −: not possible.
